# Doctors and Population

**Published:** 1898-02

**Authors:** 


### Doctors and Population.

The following table, compiled by Dr. Fanny Leake, of Austin, Texas, with great pains and care, is interesting, as showing the extent to which the doctors prevail in the United States; and also throws some light upon their relative ability to deal with disease. Those States having less than 500 to each doctor, are embraced in the upper bracket; States having one doctor to 900 or more population, embraced in the lower bracket.

TABLE SHOWING PROPORTION OF POPULATION TO THE DOCTOR—BY STATES—WITH DEATH RATE IN EACH:

| STATES. | Death Rate per 1000. | Population to each M.D. | STATES. | Death Rate per 1000. | Population to each M.D. |
|---|---|---|---|---|---|
| Me | 15.19 | 567 | N. Dak | 9.39 | 901 |
| N. H | 18.79 | 669 | S. Dak | 8.22 | 906 |
| Ver | 16.32 | 531 | Neb. | 7.99 | 663 |
| Mass | 20.15 | 555 | Mont. | 7.66 | 535 |
| R. I. | 21.18 | 636 | Wy | 6.82 | 1011 |
| Conn | 19.52 | 655 | Colo | 13.23 | 449 |
| N. Y | 20.53 | 538 | Idaho | 9.14 | 773 |
| N. J | 21.00 | 874 | Wash | 7.71 | 537 |
| Penn | 13.98 | 623 | Ore | 8.21 | 480 |
| Del | 18.53 | 705 | Cal | 14.65 | 383 |
| Md. | 17.27 | 520 | Utah | 10.19 | 818 |
| Va. | 14.03 | 837 | Ari. | 9.61 | 627 |
| W. Va | 10.85 | 617 | Nev | 9.48 | 953 |
| N. C. | 11.38 | 1191 | | | |
| S. C. | 13.46 | 1086 | States less than 500 to the doctor. | | |
| Ga | 11.25 | 909 | | | |
| Fla | 10.50 | 525 | Dist. Col. | 25.85 | 268 |
| Ala | 13.81 | 942 | Cal | 14.65 | 383 |
| Miss | 11.55 | 944 | Colo | 13.23 | 449 |
| La | 14.62 | 766 | Ore | 8.21 | 480 |
| Tex | 11.84 | 484 | Tex | 11.84 | 484 |
| Dist. Col. | 25.85 | 268 | Ind. | 11.03 | 479 |
| Ky | 12.85 | 599 | | | |
| Tenn. | 13.49 | 574 | States over 900 to the doctor. | | |
| Ark | 12.76 | 612 | | | |
| Mo | 12.16 | 565 | N. Dak | 9.39 | 901 |
| Kas | 8.42 | 645 | S. Dak | 8.22 | 906 |
| Ohio | 13.57 | 848 | Ga | 11.25 | 909 |
| Ind | 11.03 | 479 | Ala | 13.81 | 942 |
| Ill | 13.88 | 552 | Miss | 11.55 | 944 |
| Mich. | 11.95 | 561 | Nev | 9.48 | 953 |
| Wis | 11.06 | 854 | Wy | 6.82 | 1011 |
| Minn | 11.89 | 826 | S. C | 13.46 | 1086 |
| Iowa | 9.16 | 562 | N. C. | 11.38 | 1191 |

—*Ga. Jour. of Med. and Surg.*

THE total registration of members and delegates at the Philadelphia meeting of the American Medical Association was 2,158.

